# Willing and unwilling digital cyborg assemblages: University students talk about mental health apps

**DOI:** 10.1177/20552076231210658

**Published:** 2023-10-29

**Authors:** Ryan Van Der Poll, Bronwyne Coetzee, Jason Bantjes

**Affiliations:** 1 Department of Psychology, 26697 Stellenbosch University, Stellenbosch, South Africa; 2 Mental Health, Alcohol, Substance use and Tobacco (MAST) Research Unit, South African Medical Research Council, Cae Town, South Africa; 3 Department of Psychiatry and Mental Health, 37716 University of Cape Town, Rondebosch, South Africa

**Keywords:** E-mental health, mental health apps, university students, qualitative, mental health, cyborgs, techno-optimism, responsibilisationism, technoscepticism

## Abstract

**Objective:**

Explore how students talk about mental health mobile applications (i.e., apps).

**Methods:**

Data collected in focus group interviews with 51 students (all self-identifying as having mental health problems) and analysed using inductive thematic content analysis.

**Results:**

Participants describe mental health apps as an anti-dote to the loss of control, vulnerability, helplessness, impotence, isolation, conspicuousness, and shame which characterise their experience of mental illness. They describe the on-campus clinic as inaccessible and associated with “serious” problems, while configuring psychologists and psychotherapy as out of reach, scarce, formal, structured and anxiety provoking. In contrast, they imagine mental health apps as informal, relaxed, inviting, and accessible. Participants expressed openness and optimism about using apps to improve their mental health. They idealise technology as a means to connect effortlessly, anonymously, and informally, as well as learn skills, assert agency, and act responsibly. They also articulate reluctance to trust technology, show cognisance of participating in a capitalist economy, demonstrate scepticism about the legitimacy of mental health apps, and call for regulation, thereby resisting the position of responsible neoliberal subjects.

**Conclusion:**

Participants express ambivalence towards mental health apps without surrendering to either technophobia or technophilia. They express faith in technologies’ potential to support mental health while questioning the implicit assumption that people are competent to manage their own mental health. In talking about mental health apps students reproduce broader cultural discourses (including techno-optimism, techno-solutionism, somatopiamism, neo-liberalism, responsibilisationism, technoscepticism, and discourses about neuroplasticity and self-help) thus presenting themselves as both willing and unwilling digital cyborgs.

## Introduction

The use of the internet, smartphones and related digital technologies to deliver mental health information, services, and treatment (i.e., e-mental health) is increasingly ubiquitous,^1^ changing the way individuals relate to themselves, their bodies, illness, and healthcare professionals.^2^ The digital health revolution is largely seen as unequivocally good, mostly because digital technologies hold the promise of increasing access to effective healthcare, lowering the costs of providing and consuming healthcare, and giving individuals more control over and responsibility for their own health.^3^ While concern has been raised about some of the technical issues related to the reliability, safety, testing, and ethical use of digital technologies in healthcare,^3^ there has been comparatively little critical examination of how digitally mediated health interventions may affect individuals’ experiences of embodiment, selfhood, and social relationships.^2^ Critical scholars have noted that, while it is important to ask questions about whether e-mental health can achieve its promise of increasing equality and access to effective treatments, it is equally important to ask what e-mental health reveals about beliefs and practices related to the self, medicine, and culture.^1^ Understanding how digital interventions shape and are shaped by patients’ experiences of themselves, their bodies, illness, and relationships with healthcare professionals is important to ensure that technologies fulfil their potential to revolutionise healthcare for the better. The aim of this interpretive qualitative study is to explore how a group of undergraduate university students talk about mental health apps (i.e., web or mobile phone platforms that provide users with access to emotional support or tools to assess, promote, and/or restore mental health). Specifically, we aim to answer the following research questions: *What are students’ subjective experiences of mental health, traditional campus-based counselling, and mental health apps? How do students position themselves in relation to mental health and mental health apps? How do students configure the health professional and what do they understand is meant by getting help when using mental health apps?* We have focused specifically on mental health apps (hereafter referred to simply as apps) because of the rapid growth and uptake of these technologies and because these types of technologies are markedly different from other forms of digital interventions, like telepsychiatry, online therapy, virtual reality assisted therapies and wearables.

### Student mental health and the promise of apps

Globally, concern has been expressed about the poor mental health of university students and the urgent need for accessible and scalable interventions to support student well-being.^4^ Large multinational studies suggest that the 12-month prevalence of common mental disorders among first-year students may be as high as 31%^5^ while the 12-month prevalence of non-suicidal self-injury is 8.4%.^6^ Similarly, the 12-month prevalence of suicidal ideation, plan, and attempt are estimated to be 17.2%, 8.8%, and 1.0%, respectively.^7^ Left untreated these mental disorders have an enduring deleterious impact on academic and social functioning.^8^ While a range of effective treatments are available for common mental disorders,^9^ most students with mental health problems do not receive treatment.^10^ Typical barriers to accessing treatment include a lack of information, lack of perceived need, structural and attitudinal barriers, and concerns about stigma, confidentiality, and the effectiveness of treatments.^11^ Apps could be one way to overcome barriers to treatment and increase students’ access to effective interventions. Indeed, systematic reviews have described a wide range of internet- and mobile-based interventions that are effective for the prevention and treatment of students’ common mental disorders,^9,12^ suggesting that there is considerable potential to integrate apps into student counselling services. Integral to the implementation of apps into routine counselling services is a need to understand how students experience and relate to mental health problems, traditional counselling services and the use of apps, as well as how using these technologies shapes their understanding of mental health and what it means to receive mental healthcare.

### Theorising e-mental health technologies

As apps and other e-mental health solutions gain increasing traction they will continue to disrupt and reconfigure cultural practices and concepts of mental health and psychotherapy.^1^ This raises interesting questions about how e-mental health will contribute to and contest the reproduction of a cyborg, post-human mentally healthy self and how these technologies will shape users’ understandings of constructs such as “mental health”, “psychotherapist” and “psychotherapy”. While it seems likely that e-mental health will contribute to the disembodiment of mind and mental health, it remains yet unclear what the consequences of this might be for conceptualising and delivering mental health services. The work of feminist scholar Donna Haraway and the concept of the *Cyborg* may be useful to theorise how e-mental health technologies shape users’ ideas about human embodiment, subjectivity, mental illness, mental health, and treatment.^13^ Haraway argues that individuals living in technology rich environments have become cyborgs (i.e., cybernetic organisms) as a consequence of the blurring of distinctions between human and machine.^14^ Haraway asserts that there are two kinds of cyborgs, the metaphorical cyborg (i.e., a creature of fiction and the figure that contests assumptions, crosses boundaries, disrupts political systems, operates in liminal spaces and opposes convention through its hybridity) and the literal cyborg (I.e. the material configuration via the military-industrial-entertainment complex that appears as a character in science fiction films, and/or the warrior macho human-machine and/or the medicalised body).^15^ The concept of the cyborg thus operates at two different ontological levels, as both a social reality and a creation of fiction. The human body cannot be neatly categorised as one thing in static, binary, essentialist, or mutually exclusive categories. Likewise, technologies cannot be separated from humans.^16^ We can only understand our bodies/selves through technologies and our bodies/selves give meaning to technologies through the human–machine interactions of everyday life.^13^ The concept of the cyborg reminds us that our subjective body/selves are inevitably split, indistinct, contradictory, and ambiguous.^16^ As *cyborgs* we interact continuously with technologies while simultaneously participating in a culture which idealises science for its ability to save us from the messiness, suffering and disease which are an inevitable part of our fleshy bodies and fragmented minds.^15^ The cyborg concept has been used to theorise various mobile health technologies, including running apps,^17^ wearables and other sensors that gather digital health information,^18^ and health and human rights in a digital age.^19^

Lupton extended and refreshed the cyborg concept by proposing the construct “*digital cyborg assemblage*” to denote the body that is enhanced, augmented or in other ways configured by its use of digital media and digital technologies,^13^ and has applied the concept widely to theorise the social, cultural, and political dimensions of digital health technologies.^20^ Lupton's concept of an assemblage neatly denotes the idea that human bodies are complex and dynamic configurations of flesh, others’ bodies, discourses, practices, ideas, and material objects,^13^ reminding us that the use of any health technology cannot be separated from a broader political system and the influences of neoliberalism and capitalism. Both Haraway's concept of the cyborg and Lupton's concept of the digital cyborg assemblage provide useful theoretical lenses for exploring how students talk about apps as technologies to promote their mental health.

## Methods

This descriptive-interpretive qualitative study^21^ aimed to explore how a group of undergraduate university students talk about mental health apps. The research was guided by three research questions: (1) What are students’ subjective experiences of mental health, traditional campus-based counselling, and mental health apps? (2) How do students position themselves in relation to mental health and mental health apps? (3) How do students configure the health professional and what do they understand is meant by getting help when using mental health apps?

Interpretive description is a qualitative research method which is congruent with a constructivist and naturalistic epistemology.^21^ As such the approach acknowledges the constructed and contextual nature of human experience that at the same time allows for shared realities.^22^ This approach is increasingly being used in health research to explore phenomena related to health care practices and clinical phenomena related to illness and health.^23,24^

### Recruitment

Undergraduate students with a history of mental health problems were recruited via email and social media posts to participate in focus group discussions on the use of technology to support mental health, between March and May 2021. To be included in the study students had to be registered undergraduate students and self-identified as having experienced mental health problems. Email invitations to participate in the study were sent to all undergraduate students (n = approx. 20,000), with an initial email sent in March and a follow-up email sent in May. Initially, 209 students responded expressing interest or seeking additional information about the study, of which 161 met inclusion criteria and agreed to participate. These students were then invited to sign-up for one of the scheduled focus groups, but only 26 students responded and attended a focus group. A second wave of recruitment took place in May 2021 via another email invitation sent to all undergraduate students. This time 43 students expressed interest, of which only 25 attended one of the focus groups. It is not clear why there was such a marked drop-off in participation although we suspect this was related to the third-wave of COVID-19 in South Africa at the time which cause significant disruptions on campus and to students’ lives.

### Data collection

Data were collected via online and in-person focus group interviews, in which participants were asked about their experiences of mental health difficulties, campus-based mental health support services, and mental health apps. Online focus groups were facilitated via MS Teams and in-person groups were conducted in a private's space on campus at times convenient to the students. Participants could choose to either participate online or in-person; the online groups were offered to those who wanted to observe social distancing given the recent COVID-19 pandemic and/or students who wished to remain anonymous by keeping their cameras off during the focus group. Each focus group had no more than four participants to create a more intimate space where students would have the space, time and opportunity to contribute to the discussion without the anxiety of talking in a large group.

Focus group interviews were facilitated by RVDP (an experienced lay counsellor and psychology masters student) under the supervision of JB and BC (both of whom are PhD level psychology graduates). Throughout the focus group interviews, participants were asked to clarify what they were saying and to expand on things which were not clear. The facilitator also employed the technique of ‘member checking’ (asking clarifying questions to check that the facilitator was correctly understanding and interpreting what participants were communicating) to improve the data's trustworthiness.^25^ Focus group interviews were audio-recorded and ranged between 49 and 91 min. Participants were compensated for their time with a voucher valued at ZAR 50 (the equivalent of approximately $2.80 at the time of the interviews).

### Data analysis

Interviews were transcribed and the initial coding was done by RVDP using the six-step process of reflexive thematic analysis,^26^ and were then subsequently reviewed by BC and JB to gain consensus on the themes. An inductive data-driven approach was used to code the data, with the assistance of Atlas-ti software. The research questions served as an initial framework for organising the data, and provided structure to the analysis process. The research questions helped us to identify the superordinate themes, while the indictive coding of data illuminated the sub-ordinate themes. We made theoretical sense of the data once sub-themes had been identified. The themes identified are presented below with verbatim quotes to illustrate each theme and enhance the credibility of the findings. A range of quotes from different participants across all focus groups have been included to guard against bias and the selective use of interview data. Pseudonyms have been used to protect participants’ privacy. We used the *Consolidated Criteria for Reporting Qualitative Research* (COREQ) to improve the quality and transparency in the reporting of the methods and findings.^27^

### Ethical considerations

Ethical clearance for the study was obtained from the Research Ethics Committee (Social, Behavioural and Education Research, Reference number REC-2021-15506) at Stellenbosch University. All participants provided written informed consent prior to data collection. All data have been de-identified and securely stored on a password-protected cloud-based server.

## Results

The sample consisted of 51 undergraduate university students interviewed across 15 focus groups. All participants were undergraduate students completing their first degrees and were thus typically between 18 and 21 years old. Most participants self-identified as female (n = 37) and were in their first (n = 21) or second (n = 22) year of study. All participants were enrolled at a well-resourced university that has free campus-based counselling services provided by registered psychologists. The student counselling centre provides individual and group interventions, and there is typically a short waiting list of one to two weeks to secure an appointment. As reflected in Table 1 and discussed below, three superordinate themes were identified, each of which had several overlapping subordinate themes.

**Table 1.** Overview of supraordinate and subordinate themes.

Superordinate themes	Subordinate themes
Subjective experiences of mental health and illness	Control Competence Conformity Concealment Connection
Experiences and perceptions of campus-based student counselling services	Inaccessibility of series Low expectation of receiving help Counselling services are formal, structured and only for serious problems Anxiety provoked by making their vulnerability visible.
Experiences of e-mental health	Openness and optimism Better access to mental healthcare Fantasy of being able to connect anonymously and informally Using technology to learn skills, assert agency and act responsibly Scepticism.

### Subjective experiences of mental health and mental illness

Participants spoke frankly about their lived experience of mental health problems, which they said were explicitly tied up with five subordinate themes: (1) Control; (2) Competence; (3) conformity; (4) Concealment; (5) Connection.

Participants equated mental health problems with losing control, which engenders feelings of fear, impotence and helplessness while also threatening participants’ sense of agency and competence. They used words like “*spiralling*”, “*falling*”, “*loosing balance*” and “*overwhelmed*” to denote their experience of losing control. Sean explained:

*And then you do sort of lose your balance and then you fall.*

And Tessa said:

*…. it can get to an extent where you have zero control over it (everyday life), and how to go about it.*

Participants describe how the loss of control extends to many areas of their lives including an inability to control their feelings, thoughts, and actions, which makes it difficult to accomplish goals and complete tasks. Kabelo articulated his struggles to control his thoughts, saying:

*… there were too many thoughts, and I really couldn’t focus … too many thoughts were clouding my mind.*

Participants describe a fear of being seen as incompetent and a reluctance to ask for help because doing so would signal failure. Kaya explained:

*… sometimes it's just too much, you don’t want to turn to your parents or your friends or something because you don’t want to seem like a failure.*

And Ayesha said:

*… a lot of people are just afraid of like admitting, ‘Yes, I struggle with my mental health’ because they feel they are going to be seen as less competent.*

Participants explicitly talk about “*struggling*” (with all its connotations of fighting and wrestling), and they imply that, unlike most other students, they are either losing or feeling wounded by the battle. Furthermore, they say that the erosion of their sense of control and agency sets them apart from their “mentally healthy” peers, who appear to be in control and able to cope with the struggles of university. Evelyn explained:

*… everybody is supposed to be coping. … and you don’t want to be that one person that is not.*

Participants said that they are expected to be able to cope on their own and articulate a desire to conform to this norm. Failing to conform leaves them with feelings of shame. Ayesha said:

… *there is a lot of stigma on the campus …. the kind of attitude there, is, “Yes, everybody is struggling so if we can struggle through it, then why can’t you?*

Participants speak of mental illness as something stigmatised, shameful, and embarrassing. They describe how they work to conceal their feelings and hide themselves away to keep up the appearance of coping and avoid judgement. Diana said:

*I don’t want everybody to know about my problems. They’re my problems … it's embarrassing to tell people.*

And Camila said:

*…. (there is) shame involved in admitting, even to yourself, that you have an issue.*

Similarly, Illiana used the words “*frowned upon*” and “*taboo*” to communicate her perception that disclosing her mental health problem will be met with disapproval and ostracisation:

*It's frowned upon to even consider having mental health issues … it is a complete taboo.*

Participants associated concealing distress with strength, implying that showing one's feelings is a sign of weakness. Kaya explained:

*I didn’t want to talk to people around me because I wanted to appear stronger.*

But not talking to other people and hiding away (making sure they are not seen) compromises participants’ authenticity which in turn contributes to their withdrawal from and difficulty to connect with other people. Participants’ experience is that of feeling distant and disconnected from their “mentally healthy” peers. Isolation was thus an integral component of their experience of mental illness. Dawson described their experience of isolation, saying:

*… you have to get used to being on your own, doing everything for yourself, so it's definitely overwhelming to get used to …*

The capacity to retain control, deal with the blows of life, exercise agency, demonstrate competence and conform to norms were seen as defining features of mental health. Participants believed that it is normal to struggle at university and the expectation is that they should be able to cope. Failing to retain control and demonstrate competence sets participants apart from their peers and is associated with shame and a need to avoid being seen.

### Experiences and perceptions of campus-based student counselling services

Participants spoke about their perceptions of traditional campus-based counselling services and their first -hand experiences of accessing these services. Four subordinate themes were implicit in the way the described their perceptions: (1) Inaccessibility of series; (2) Low expectations of receiving help; (3) Counselling services are formal, structured and only for serious problems; and (4) Anxiety provoked by making vulnerability visible.

Almost all participants said they were informed about the student counselling centre on campus, but many of them said they were not sure how and where to access these services or what services were available. Zaydee, for example, said,

*I don’t know where the building is – but I know there's a building.*

Participants who had previously utilised campus-based services described having to negotiate their way through gatekeepers to secure an appointment with a psychologist. Nathan said:

*I personally struggled with going to Campus help, specifically because it requires having to send quite a revealing email to someone you’d never met before … by the time you actually reach a psychologist, you’ve been through at least three or four people. By that time, you’re already so anxious just because you need to speak to so many people about something that actually is quite painful.*

Participants also expressed frustration at having to wait for an appointment and seemed to have an expectation that they should be accommodated quickly if they reached out for help. Jaime explained:

*… it takes a long time to get an appointment*.

And Ellie said:

*When I contacted the student centre for counselling, it took a very long time to be able to start receiving support. … it probably took about two weeks for me to finally start seeing someone.*

Participants expressed a low expectation of receiving help from the student counselling centre. Inez, for example, said, “*I don’t know if they have, like, enough people (psychologists) there.*” and Maria said, “*I do know that they are quite overloaded.*” Nadine described their perception of the resource constraints, saying:

*… they (Student Counselling) are actually quite busy, so it's very difficult to get an appointment and … they are so full and because there are so many students, I feel there's not enough, you know, there's just not enough counsellors to accommodate all of us because we’re a really big group*.

Participants used words like “*serious*”, “*formal*” and “*structured*” to describe their perception of seeing a psychologist for traditional face-to-face therapy. Ayesha explained that going to the student counselling centre implied that “*she's got big issues”,* which led her to believe that it was only for “*serious problems*”. Similarly, Jade explained that going to counselling was a “*big thing*” that elicited anxiety before going on to say:

*If I go (to student counselling) I must be structured, I have to be formal.*

The formality and seriousness associated with counselling precipitated feelings of anxiety and discouraged students from using the services. Aligned to this they described how uncomfortable and anxiety provoking it was to sit in front of someone, subject themselves to the gaze of a psychologist and use their voices to talk about their mental health problems. Diana explained:

*… (it's difficult to) sit in front of someone and talk to them about it.*

And Zara said,

*I’ve had moments where I don’t think I can tell someone to their face, “Well, this is what I’m going through,” because the eye contact just causes anxiety.*

While Kabelo said:

*it's difficult for some people, I know it's also difficult for me at times, to be vulnerable and say; “Oh, this is what I’m going through, this is why.”*

While participants are aware that free student counselling services are available on campus, they perceive several barriers to accessing these services including lack of information about where and how to access services, low expectations of receiving help, and a perception that they will have to wait a long time to be helped. They articulate a perception that counselling services are formal, structured and only for “*serious problems*”. For the participants in this study receiving therapy is associated with making yourself seen and allowing yourself to be vulnerable, both of which elicit feelings of anxiety as well as fear of judgement.

### Experiences of using apps to promote mental health

Participants said that they had first-hand experience of using mental health apps, including meditation and mindfulness, journaling, and sobriety (i.e., substance use tracking) apps, as well as apps that facilitate on-demand online therapeutic support from “real” listeners in one-on-one real-time chat. There were five subordinate themes implicit in the way they spoke about these apps: (1) Openness and optimism; (2) Better access to mental healthcare; (3) Fantasy of being able to connect anonymously and informally; (4) Using technology to learn skills, assert agency and act responsibly; (5) Scepticism.

#### Openness and optimism

Participants agreed that the use of apps to promote mental health was commonplace and expressed a perception that peers were already accustomed to using these technologies. They said the move towards greater use of apps was desirable and inevitable. Jade explained:

*… mindfulness apps and gratitude apps and like journaling apps are very mainstream, and a lot of people just use them anyway.*

Participants with experience of using mental health apps, said they found it helpful and therapeutic, even if they had initially been sceptical. Illiana, for example, said:

*It was quite helpful.*

While Hariet, said:

*I was like, “Oh ok, it's not really going to work, but I’ll try it anyways.” … I tried it for about two weeks, and after the first week I just slept so much better.*

Participants who had not previously used apps, expressed an openness to utilising them in the future and optimism that apps could be helpful, as reflected in comments such as,*“I definitely think apps would be quite helpful”* (Delilah) and “*I definitely would not mind seeing if such a thing is possible. I definitely think it would do so much good”* (Nathan). Even participants who preferred face-to-face contact for emotional support acknowledged that apps could be a helpful adjunct to existing services and support systems: Joshua said:

*I think like even as someone who prefers to have someone to talk to, having any support at any point is very helpful. Like if I’m unable to contact the people I would normally talk to, because it's like the wrong time of day for them, or I don’t want to be a burden for someone in that moment, then it will be very useful to have some sort of support or somewhere to go.*

Not all participants had completely positive experiences and a small group of them spoke about the difficulties of finding the “right app” (i.e., an app that matched their specific needs and preferences) especially in the absence of professional guidance. Participants also spoke about the effectiveness of apps being short-lived. Abigail explained:

*(The app was) good for the moment but then after a month, it's just not working anymore.*

Despite the handful of negative experiences, the over-riding sentiment was one of optimism and hope for what apps could offer. Indeed, as we will demonstrate in the themes below, there was an overriding perception that apps could increase access to mental health services, alongside a strong tendency to idealise apps as technologies that enable users to effortlessly and anonymously connect to other people with similar experiences as well as acquire helpful self-management skills.

#### Better access to mental healthcare

All participants said they could see the potential for apps to increase access to support, by making services easier, quicker, more convenient and less time consuming to utilise. They spoke about the comfort and ease with which apps could be used from anywhere at any time. Participants articulated a perception that it requires effort (and hence some determination, persistence, and energy) to seek help from a mental health professional, but technology could overcome this by making access comparatively effortless and immediate. Nathan said:

*it's the easiest first step for someone to go to the app – that's definitely true.*

And Rachel said:

*… a lot of people they like, find it difficult to reach out. If they could do it from the comfort of their own phone or their laptop that or just it's very minimal effort to go to a website and reach out like that. […] It's much more accessible for people if they can access an app …*

Participants expressed a fantasy that apps make support services available from anywhere at any time, and that consequently help was always close at hand. A perception was articulated that apps could give users control over when and where they access help. Graham explained:

*… because it's easier to just go on an app whenever you feel like you need to access resources.*

And Sadie said:

*The idea of having an app where you can basically access all of the resources is much more helpful to me …, my phone is everywhere with me so if I’m struggling with something now, I can access it in the moment.*

Participants took comfort from their belief that apps could give them immediate access to help “*in the moment*”, “*after-hours*” and in an “*emergency*”, making it seem as if help (as Sadie expressed it) “*is always with me*”. Nompelo explained:

*I know especially for me, it's usually after hours, because during the evening you have more time to think and more time to overthink and then things just get bad. So if at 9 in the evening, I can like get access to an app, that would be absolutely amazing.*

Participants spoke of apps being beneficial because of the immediacy with which these technologies could be used to access support afterhours when other support services were inaccessible. Demi explained how technology could relieve “pressure” and thus serve as a balm, until other services can be accessed:

*… having the app, I think, would relieve some of the pressure until you actually go see a psychologist ….*

While Mandla explained:

*The app makes it easier to get in touch with someone quicker.*

Participants explained how anxiety and depression sometimes made it difficult to exercise agency and made them reluctant to leave home. By seeing the use of apps as effortless and immediate, they express a fantasy that technology makes it easier to get help. Kristin said.

*But also, if you’re anxious or really depressed or just like, something really bad's happened, you might not even want to leave your room. You might not want to shower and so it's nice to have something that is right next to you. Because let's be honest, we all carry our phones on us everywhere. But I mean like it's right there, it's two seconds to access.*

Participants expressed a desire to get help without having to leave home. They spoke of the convenience, comfort, time-efficiency, and emotional safety of being able to access support at home. Jade said:

*And that's what made it easier for me to speak to someone […] I could just like be in my room, and for me, my room is my safe space, so it becomes much easier to like share because I know it's homey, like I don’t have to feel like, okay, I have to be structured, I have to be formal, so that made it a lot easier as well as the accessibility.*

Dana explained how using e-mental health was time-efficient, saying:

*I would think that most students would prefer it because it is also quicker. You can do it at any time, you don’t have to make an appointment with someone. You don’t have to spend fifteen minutes walking or driving to the appointment, sitting there for an hour and then coming back again.*

Participants articulate a fantasy that apps give them control of when and how they receive help, that they are never alone and that their emotional needs can be met immediately and without struggling or expending energy. They draw comfort from the belief that apps make it easier, safer, and more convenient to access help compared to traditional campus-based counselling services.

#### Fantasy of being able to connect anonymously and informally

Participants spoke about apps as technologies that could be used to connect anonymously and informally with other people. They articulated a fantasy that apps could help them achieve connectedness and closeness to other empathetic people, while also maintaining distance, privacy, autonomy and remaining invisible. Indeed, the theme of being connected without being seen was prominent throughout the focus groups. Jade described their experience of using an app that connects users to “listeners” via real-time on-demand chat:

“*(The app is) quite nice because it has a lot of people using it, so there's always someone you can talk to … it's quite nice to kind of talk to someone completely anonymously and you can just kind of tell them how you feel and having that empathy response is quite nice, […] I think that was quite helpful, and I like that you could filter certain things, so that you actually have someone who knows what you’re talking about.*

Jade's use of the words “*there is always someone you can talk to*” points to their phantasy that apps can be a conduit for magically finding connections at any time with others “*who know what you are talking about.*” Furthermore, Jade's belief in the apps’ ability to satisfy their desire to remain unrecognised, hidden, and faceless is reflected in the words “*talk to someone completely anonymously.”* It is apparent that Jade believes that apps can facilitate self-disclosure, as was also articulated by many other participants. Participants said that by facilitating connections, apps helped them to feel part of a bigger supportive community, thus facilitating a sense of belonging. Tessa described how appscan “*create a little community*” of people with shared experiences, and Layl spoke of turning to an app to find a “*supportive environment*”.

Participants articulated a perception that apps preserves anonymity and privacy. Camilla explained how apps could overcome the stigma of accessing professional treatment:

*…. one of the real benefits of an app is that no one really needs to know that you’re on the app, like I think there's a lot of shame involved in admitting, even to yourself, that you have an issue. And then it's like you just get an app, nobody needs to know that you have the app, and then you can get on the app and be okay, cool, no-one knows that I go see someone, and then you kind of get into it.*

And Claire said:

*it (using an app) is definitely more like a personal thing, […] no one else has to know.*

Participants spoke about how apps helped them to express their vulnerability and say things that they would not otherwise be able to say. Kabelo explained:

*I think it (using an app) is easier because it's difficult for some people, I know it's also difficult for me at times, to be vulnerable and say; “Oh, this is what I’m going through, this is why.” Whereas it's easier for me to speak to an app, right, as much as they don’t talk back, then I know there's no things like judgment or insecurity that I’m feeling.*

Participants described the catharsis of being able to speak freely and said that apps facilitate self-expression. Joshua explained:

… *just talking about something so that you don’t bottle it up and it just explodes at a later date … You can just go to the app and talk to the person it doesn’t even need to be a qualified person because sometimes you are stuck in a situation where you just need someone to talk to and you don’t have anyone at that time.*

And Tyler said:

*Speaking to people helps so much, realising that you are not the only one going through what you’re going through, and then also like learning through other people's experiences, it's really really helpful.*

One participant (Nathan) used the metaphor of a bridge to explain how apps help them to connect to other people in cyber space:

*… an app would definitely help, bridge that kind of uncomfortable silence between people.*

The idea that apps can overcome “*uncomfortable silence between people*” points to Nathan's phantasy that apps can help them establish connection and intimacy in ways that are not normally possible while also reducing the anxiety of being separate from others in physical space.

The idea that apps can facilitate disinhibition and create freedom for users to express themselves was very common throughout the focus groups. This perception was also linked to the idea that apps could help users overcome barriers – not only barriers to accessing help but also barriers to self-disclosure. Vivian said:

*… (when using an app) there would be no barriers, I wouldn’t want to hide anything.*

And Graham explained:

*… having an app lowers the barrier of entry.*

Vivian and Graham's description of technology dismantling barriers points to the idea that apps helps open space (at least metaphorically and psychologically) which gives people freedom to move in ways that don’t feel possible in physical spaces and other areas of their lives. In this sense, apps are positioned as liberating while also enabling authenticity. Diana explained:

… *it (the app) kind of gives me, you know, that bit more space, and I can really say what I feel.*

And Jaime said:

*… you can express yourself better that way. If you’re crying, or completely overwhelmed, I can’t get words out. So, it is a lot easier to type.*

Other participants contrasted their experiences of using apps with their experiences of seeing a therapist face-to-face, saying it was easier for them not to “face” someone when asking for help or showing vulnerability. Being behind a screen offered protection and decreased anxiety which made it easier to talk freely. Dana said:

*It's easier to sit behind your screen and type into your journal how you’re feeling, than actually going to someone and being like, “This happened” or “I’m feeling this way”. So it's definitely easier not to sit in front of someone and talk to them about it.*

Zara elaborated by describing how the disembodied experience of accessing help in cyber-space facilitated disclosure and made it easier to be vulnerable:

*With chatting, you don’t show your body language, what you’re feeling on your face, and so it becomes easier to say whatever and then let it be. Because I know I’ve had moments where I don’t think I can tell someone to their face, “Well, this is what I’m going through!” Because the eye contact just causes anxiety. When just chatting to someone (on an app), I just type it all out, send, and once it's sent, it's sent. Then there's nothing I can do afterwards, which makes it almost a bit easier to be vulnerable. Because now you don’t have someone looking at you which makes you feel a bit more scared which can cause you to close-up*.

For Zara, the sense of distance and space created by using an app made them feel less anxious (i.e., safer) and hence more at ease to reveal themselves. When Zara says, “*I just type it all out, send, and once it's sent, it's sent.”* she is communicating something about the catharsis she feels at being able to express something personal and then immediately feel distance from it because it is “sent” and hence no longer “here” with her. Zara's description of the anxiety of being completely seen and the discomfort of eye contact when disclosing sensitive information in person, was echoed by other participants who expressed a desire to be able to control how much of themselves they revealed.

Participants spoke about the appeal of informality when using apps, saying that using these technologies felt less structured, and more relaxed, natural, and commonplace compared to consulting a professional in person. Ayesha explained:

*I feel like it's almost like less formal if you use an app, people would see it as a less formal kind of intervention, so it's not like a, oh, she has to go to this office because she's got big issues whatever, it's like I feel it could become a more common thing …. I feel I can just bring it down to a more normal person level instead of, like oh, she's got a problem, where it can become more common practice to use the app than like an abnormality where now less pathology where she has to go to counselling.*

And Claire said,

*What I love about these apps is the informality about it, because it just takes a lot of pressure off of you.*

Kabelo added:

*… (using apps) makes everything more chilled and relaxed and people, I think, people tend to open up or speak about their feelings more when they are relaxed and comfortable.*

Claire Ayesha and Kabelo describe how the informality of using apps reduces their level of anxiety, making the process more relaxed, comfortable, and conducive to talking about emotions. Furthermore, Ayesha's use of the phrase “*more normal person*” and “*less pathological”* points to their perception that the use of apps makes them feel more “normal”.

Implicit in participants’ words is that part of the appeal of apps is its capacity to facilitate anonymous connections with other people, reduce the anxiety of facing someone, create space for them to express vulnerability and disclose feelings while controlling how much of themselves they reveal, as well as normalise the experience of accessing support. They position apps as devices that regulate closeness and separateness from others, by creating connections while also staying faceless and unseen.

##### Using technology to learn skills, assert agency and act responsibly

Participants said that apps could be used to learn skills that supported their mental health. They position apps as technologies that help them to learn to manage themselves and hence realise self-mastery while also being empowered. Hariet, said:

*I learnt like breathing techniques and stuff like that just to kinda help me calm down.*

And Kabelo explained:

*… I learnt how to actually do it (be mindful), so that app was, kind of, like a teaching step for me.*

The experience of feeling empowered by using an app is further reflected in participants’ assertion that when they are using apps they are “*doing it for themselves*”, which reminds them that they are independent, autonomous, and capable. Kabelo explained:

*… I think the thing with apps, which it might be, especially the positives I guess, is that you kind of can do it yourself.*

And Caleb said,

*…. at least you are doing something and you’re not just allowing your circumstances to eat you up from the inside.*

Caleb's use of the words “*doing something*” and “*not just allowing*” speak to the sense of agency and mastery they feel when using apps. By using apps, participants believe they are acting responsibly which further feeds their sense of agency. Layla articulated this, saying:

*… (apps) help you take some responsibility for your own self-care.*

Evelyn explained:

*It (using an app) also makes you feel that you are in control ….. (when you are mentally unwell) you don’t feel like you’re in any control, you’re losing more control as you are spiralling. So, if you have control of at least picking that (app), this is how I am going to move forward, even if it doesn’t work, you’ll try something else later. At least you have the choice.*

##### Scepticism

While participants were optimistic and hopeful about the possibility's apps could provide, they also expressed suspicion and scepticism about the authority and legitimacy of these technologies. Participants showed an acute awareness that apps were commercial products and that producers of these technologies might be driven by profit motives rather than altruism or any professional code of conduct. Furthermore, participants recognised that apps might not be produced or endorsed by mental health professionals. Maria said:

*if it is an app, I don’t know who made it, I don’t know if anyone is actually endorsing it if it's actually going to do any good for me.*

And Natalie articulated scepticism saying they were “*quite sceptical of them (apps),”* before going on to say:

*… it's an app, but, made by who? What are their qualifications? What gives them the right to tell me how to better myself? It's not very trust-inducing, because it just feels like it's coming from some random person.*

Participants spoke about the importance of being able to “*trust*” and “*count on apps*”, which they said was difficult when apps were not produced or endorsed by mental health professionals. Claire explained:

*… (I) already feel better about the app if mental health professionals help in the development of it, because then I know I can count on it more.*

And Irena, said:

*I think like the general idea of trust would be the biggest issue. Like first of all, like being about it being run by professionals, but also like just the confidentiality of it.*

Participants showed cognizance that by using apps they were participating in a capitalist economy as consumers of a product, and that their goals as users of the product might not be completely congruent with the profit motives of app producers. Delilah said:

*I have used a few, but the issue with them is that they all require money … the things that are offered are very limited without you buying like a subscription, so quite frustrating because obviously, I personally don’t have the money to spend on it so it's quite difficult sometimes because the things that are offered on the free platform are very limited.*

And Illiana reflected on the pricing of apps and their reluctance to want to pay for psychological help:

*Something that I’ve noticed that really actually frustrates me is that there are so many where they expect you to pay some ridiculous fee just to get the help that you need and for me, obviously, I understand if it's a psychologist and that's how they make their living. But I mean, it's an app. It's an AI thing that's trying to help you, like it really shouldn’t cost you that much, I mean, I don’t know, for me that just puts me off the whole idea of having an app.*

Participants further demonstrated awareness that apps were commercial profit-generating products by referring to the placing of advertisements in “free” apps. Joshua explained:

*… when you’re in a crisis, you don’t want to watch an ad before you solve your crisis.*

Some participants were more vociferous about their scepticism towards apps, referring to them as a distraction and as devices which only created an illusion of providing help. Camila called the use of apps a “*trap you’re falling into,*” and went on to say:

*… in my sort of limited understanding of it, the app kind of acts as a guise for actually helping yourself.*

Camelia's use of the word “*guise*” and “*trap*” suggest that they associate apps with deception and a fear that they prevent people from getting the help they really need.

Participants acknowledged that virtual spaces (like online communities and online chats) could also have the potential to do harm and become toxic environments. Caleb explained:

*Some online spaces do tend to get more destructive than they are positive.*

Layla echoed this perception by underlying the importance for regulation and control over apps, saying.

*It is very important to have something like that monitored; it's just safer for everyone.*

By acknowledging the need for regulation and by pointing to the potential for harm, participants not only de-idealise apps, but they also explicitly call for regulatory authorities to provide protection and control.

## Discussion

The aim of this interpretive qualitative study was to explore how university students talk about mental health apps. Our analysis of the data shows how participants present themselves as both willing and unwilling *digital cyborg assemblages*.^13^ They articulate techno-optimism but also express scepticism towards apps. They see technology and apps as inextricably part of them, but also believe they are separate from technology and can use apps as tools to direct their own self-actualisation. As users of apps they happily assume responsibility for their own mental health, willingly positioning themselves as good responsible neo-liberal subjects. But they also resist neo-liberalism. In adopting these multiple (sometimes contradictory, ambiguous, and ambivalent) positions towards technology they embody Haraway's concept of the *metaphorical cyborg* (i.e., the ambiguous figure that defies binary classifications, crosses boundaries, disrupts political systems, is comfortable in liminal spaces, and embraces hybridity).^15^ In the discussion below we explore these explicit contradictions and show how through the way they speak; participants reproduce broader cultural, political, and medical discourses about health and selfhood.

Participants describe how mobile digital technologies are “*always with them*” positioning technology as part of their everyday world, their usual habits and typical mode of operating. Participants see the expansion of mental health apps as inevitable, and they are both open to and optimistic about using these technologies. They articulate a utopian fantasy that apps could give them near-effortless access to psychological support anytime and from anywhere, thus imagining apps as an ever-present always accessible psychological support system. Furthermore, they idealise apps for their imagined capacity to empower them with new skills, liberate them to express themselves freely, and connect them to other people, all effortlessly and from the comfort of their own homes. Participants thus present themselves as *techno-optimists*^28^ and reproduce the discourse of *techno-solutionism* (i.e., a naïve belief that technology can solve any problem).^29^ Participants’ openness to using apps to support their mental health is perhaps unsurprising given that they belong to a generation that has been called digital natives and have been socialised into using digital tools in many other areas of their life.^30^

Participants see apps as an anti-dote to the loss of control, vulnerability, helplessness, impotence, isolation, conspicuousness, and shame that they associate with mental illness. For them, mental health is inextricably tied up with ideas about self-control, competence, conformity, and connection, all of which they believe technology will enable them to regain. Participants configure themselves as willing *digital cyborg assemblages,*^13^ who are comfortable merging with digital technologies to enhance their health. However, participants also speak of technology as a tool which is both separate from them and under their control. They position themselves as active agents who can direct e-mental health gadgets and harness technology in the service of their own well-being. Crucially participants think of apps as a tool for self-mastery and self-management, positioning apps as a self-enhancement technology that can be employed as a means of disciplining an unruly brain with its out of control and unregulated thoughts and feelings. Participants reproduce a *somatopian* discourse in which attainment of a perfect disciplined body is the ultimate postmodern utopian telos.^31^ Somatopia is the technoutopian idea that bodily perfection will be realised as soon as we have enough technology, information, self-monitoring and self-treatment, and manifests as the collective expression of privatised and personalised projects to achieve the perfect body.^31^ Somatopia is driving the rapid growth of direct-to-consumer digital health technologies,^31^ and seems to be part of the worldview of our participants.

Our participants’ assertion that apps can facilitate an enhanced self is broadly aligned with Haraway's notion of the *literal cyborg* (I.e., the material configuration via the military-industrial-entertainment complex).^15^ However, our participants’ do not configure themselves as the typical “warrior macho human-machine” that is associated with Haraway's literal cyborg.^13^ Instead, they present themselves as achieving an enhanced more authentic self by asserting agency over technology rather than as merging with technology. In other words, participants see apps as a means to realise the self by metaphorically standing on the head of technology, as opposed to transcending the self to achieve posthuman status through union with technology.^32^

Participants conceptualise apps as a bridge that can close the gap between them and other people and as a conduit for establishing connections without having to expose themselves to the gaze of others. Apps are configured as conduits for connection while simultaneously giving the user control over the level of intimacy and the degree of vulnerability they express. These technologies thus allow users to regulate the balance between closeness and separateness which seems to make it easier for them to feel safe, express themselves and achieve catharsis.

Participants express a fantasy that apps will give them quick and easy access to help without having to leave the house. They seem entirely comfortable (if not welcoming of) a technology that “brings the clinic into the home”.^33^ They describe the clinic on campus as a space that is difficult to access, associated with “serious” problems, and guarded by gatekeepers. Similarly, they configure psychologists and traditional psychotherapy as out of reach, scarce, formal, and structured. By contrast, they imagine mental health apps as informal, relaxed, welcoming and accessible; a “homely” image that stands in stark contrast to the hard, cold, metallic caricature of the robotic inhuman machine of science fiction.

Importantly, participants demonstrate an implicit belief that the “self” is the cite of mental health problems and the target of mental health apps, thus reducing mental illness to the micro, individual level. As Lupton notes, “Such approaches do little, therefore, to identify the broader social, cultural and political dimensions of ill-health”.^2^(p239) Indeed, this critique of apps as implicitly locating psychopathology within individuals is true of most conventional forms of psychotherapy that assume that change and growth toward mental health comes from within the individual.

In expressing optimism about and a willingness to use digital technologies to support their mental health, participants assume the role of “good citizens” and participate in the expansion and intensification of a neoliberal orientation to individual “responsibilisation” of healthcare,^34^ while also rendering themselves competent to manage their own mental health.^1^ Beyond this, they buy-into the idea that mental health is within one's own control^1^ and thus participate in neuroplasticity discourses (i.e., an increasingly prevalent idea in medicine and popular culture that the brain is mutable across the whole lifespan, open to insults, nourishment, and reshaping).^1^ They believe apps will help them reshape their own brains without the need for input from a mental health professional, thus configuring their brains as a “site of choice, prudence, and responsibility for each individual”.^35^

Interestingly, our participants also present themselves us unwilling digital cyborgs, by articulating some reluctance to completely trust technology, showing cognisance that they are participating in a capitalist economy, demonstrating scepticism about the legitimacy of apps, and expressing suspicion of app producers’ motives. They resist being positioned as consumers of mental healthcare and explicitly ask for regulation and control to create safety, and the endorsement of “professionals” to create trust. By calling for regulation and the participation of mental health professionals in the production and endorsement of apps, participants resist the position of responsible neoliberal subjects and question the implicit assumption underlying much of the e-mental health hype (i.e., that each of us is competent to manage our own mental health without the need for input from a mental health professional).^1^

This study has several limitations, including the fact that participants were only recruited from one well-resourced university. The use of focus group interviews may also have limited participants’ willingness to share sensitive information or express minority opinions and may have discouraged some students from participating in the research. If it is true, as our participants say, that mental health problems are stigmatised and that young people are reluctant to be seen as having problems, then individual interviews may well have been a preferable way to collect information on this sensitive topic. Importantly, data were collected in the wake of the COVID-19 global pandemic during which time social distancing and other mitigation strategies restricted students’ ability to socialise with peers face-to-face and forced them to make greater use of technology to interact with peers and attend classes online. This may have profoundly influenced participants’ experiences and attitudes toward digital technologies like apps. Nonetheless, the study provides insight into how some young people think about mental health apps and lays the foundation for similar work in other contexts and using different research methods.

## Conclusion

In the way participants speak, they explicitly acknowledge the potentialities of mental health apps but also apps’ limitations. They express ambivalence towards e-mental health without giving in entirely to either technophobia or technophilia, but nonetheless, express some faith in technologies potential to meet mental healthcare needs. It remains to be seen how misplaced their faith in technology is, but it is clear that in the way that they talk about apps and the self, they reproduce broader cultural discourses (including techno-optimism, techno-solutionism, somatopiamism, neo-liberalism, responsibilisationism, technoscepticism, and discourses about neuroplasticity and self-help) while also presenting themselves as both willing and unwilling digital cyborgs. These findings provide insight into how a group of young adults talk about mental health apps and mental illness, and how this talk reproduces broader cultural narratives.
